# Thouless pumping in disordered photonic systems

**DOI:** 10.1038/s41377-020-00408-2

**Published:** 2020-10-19

**Authors:** Alexander Cerjan, Mohan Wang, Sheng Huang, Kevin P. Chen, Mikael C. Rechtsman

**Affiliations:** 1 Department of Physics, The Pennsylvania State University, University Park, PA 16802 USA; 2 Department of Electrical and Computer Engineering, University of Pittsburgh, Pittsburgh, PA 15261 USA

**Keywords:** Nanophotonics and plasmonics, Nanophotonics and plasmonics

## Abstract

Thouless charge pumping protocols provide a route for one-dimensional systems to realize topological transport. Here, using arrays of evanescently coupled optical waveguides, we experimentally demonstrate bulk Thouless pumping in the presence of disorder. The degree of pumping is quite tolerant to significant deviations from adiabaticity as well as the addition of system disorder until the disorder is sufficiently strong to reduce the bulk mobility gap of the system to be on the scale of the modulation frequency of the system. Moreover, we show that this approach realizes near-full-unit-cell transport per pump cycle for a physically relevant class of localized initial system excitations. Thus, temporally pumped systems can potentially be used as a design principle for a new class of modulated slow-light devices that are resistant to system disorder.

## Integrated optics: combating disorder

Research into the effect of temporally-modulated pumping in coupled optical waveguides may aid the design of future on-chip slow light devices. The result could be designs which are more robust against disorder and fabrication imperfections. Alexander Cerjan and coworkers from Pennsylvania State University and the University of Pittsburgh in the USA studied adiabatic pumping, also known as Thouless pumping, in a one-dimensional array of laser-written evanescently-coupled waveguides in borosilicate glass. Nearly quantized topological transport of light was observed, even in the presence of disorder which was introduced by a variation in the spacing between neighboring waveguides. The adiabatic pumping was achieved by dynamically modulating the refractive index of the waveguide array (where temporal modulation was mapped to spatial variations). Future research will explore the connection between Thouless pumping and dynamical quantum ratchets.

## Introduction

Adiabatic charge pumps^1–4^ are an important class of topological systems, as they can exhibit robust transport and provide a physically intuitive mathematical mapping between a one-dimensional system and the intrinsically two-dimensional quantum Hall effect^5^. Moreover, photonic realizations of such systems represent alternatives to two-dimensional photonic topological insulators with chiral edge states^6–17^ for achieving compact on-chip slow-light waveguides^18–28^. In the traditional electronic picture of adiabatic charge pumps, a system with a uniformly filled valence band is periodically modulated such that after a complete cycle, each localized Wannier state in the occupied band has adiabatically evolved to be transported by exactly one unit cell. If the system is finite, then the Wannier state that arrives at the edge of the system, which cannot be pumped further, is instead pumped across the bandgap to the conduction band^1^. These systems were originally proposed by Thouless^1^ and can be viewed as Chern insulators in 1 + 1 dimensions^29^, in which the periodic modulation in time is substituted for the second spatial dimension. The parameter that defines the Hamiltonian at any given time, called the pump parameter, maps to a momentum in the corresponding 2D system that is perpendicular to the direction of pumping.

Previous studies of adiabatic pumps in optical systems have focused on observing edge-to-edge transport, in which the system is initialized in a topological edge state of the one-dimensional system and this state is evolved through a complete pumping cycle, transporting it to the opposite edge of the system^13,30–32^. Achieving this form of topological pumping requires ‘state-level’ adiabaticity; i.e., the modulation of the system does not introduce any coupling between states that reside within the same bulk band due to deviations from perfect adiabaticity. If such couplings exist, then many bulk bands will be populated during the pumping process, and the wavefunction will not be completely pumped to the opposite side of the system. This constraint is limiting, as the spacing between the individual states that constitute the bulk bands of the system is inversely proportional to the size of the finite system, effectively constraining this procedure to small system sizes or long modulation periods. In contrast, in this work, we focus on *bulk transport* in adiabatically pumped optical platforms, in which only bandgap-level adiabaticity is necessary to achieve quantized pumping due to the conservation of lattice momentum. In previous theoretical studies in the context of electronic pumps^33–36^, it has been demonstrated that deviations from adiabaticity in Thouless pumps lead to corrections to the transport of the wavefunction which are only polynomially small in the driving frequency, rather than exponentially small. Experiments in cold-atom systems have probed bulk topological pumping^37,38^, but here, we also seek to address the effects of disorder, as this breaks translational symmetry, implying that the lattice momentum is no longer conserved, and thus potentially degrades the bulk transport properties^2,3,39,40^.

In this article, we experimentally demonstrate nearly quantized topological transport in Thouless pumped optical systems that only possess bandgap-level adiabaticity and are initially excited using single-site excitations. We find that the addition of disorder does not significantly affect the observed transport until the strength of the disorder reduces the size of the mobility gap of the instantaneous spectrum to be approximately equal to the modulation frequency, i.e., when Zener tunneling can occur. Our experimental system is fabricated in an evanescently coupled one-dimensional single-mode waveguide array using femtosecond direct laser writing^41^. Within the waveguide array, we are able to achieve adiabatic pumping by modulating the index of refraction of the waveguide arrays as well as the relative distance between neighboring waveguides, while system disorder is fabricated into the arrays by changing the average spacing (over one pump cycle) between neighboring waveguides.

## Results

**Fig. 1:**
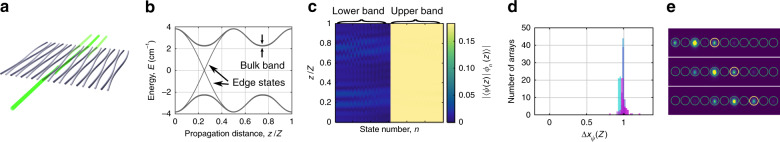
**a** Schematic of the Rice–Mele waveguide lattice. **b** Instantaneous eigenvalue spectrum for a waveguide array designed to exhibit the Rice–Mele model, Eqs. (2)–(4), over the full adiabatic pump cycle. **c** Spectral decomposition of the wavefunction over the instantaneous eigenstates of the ordered system with periodic boundary conditions, |*φ*_*n*_〉, as a function of the propagation distance. The simulated supercell contains 30 unit cells. The instantaneous eigenstates are ordered by their energy, so all of the upper band (i.e., occupied) states of the system are shown on the right half of this plot. **d** Histogram of the experimentally observed displacements of the waveguide array as light is injected into the bulk of the system one waveguide at a time. Different colors represent different incident wavelengths, *λ* = [1450, 1500, 1550, 1600, 1650 nm], listed in order of cyan to magenta. **e** Three exemplars of the direct observation of transport at the output facet of the waveguide array at *λ* = 1650 nm. The waveguide locations are outlined in green circles, except for the location of the initially excited waveguide, which is outlined in yellow

Our experimental system consists of an array of evanescently coupled single-mode waveguides that are laser-written into borosilicate glass. As the diffraction of light through waveguide arrays is governed by the paraxial Schrödinger equation and the light is tightly confined to the waveguides, this system can be modeled using the tight-binding approximation, with the propagation distance along the waveguides, *z*, replacing time evolution,

1 $$i\partial _z\left| {\psi \left( z \right)} \right\rangle = \hat H\left( z \right)\left| {\psi (z)} \right\rangle$$

where |*ψ*(*z*)〉 is the envelope function of the electric field on each of the waveguides for a given incident wavelength, *λ*. Moreover, we will refer to the eigenvalues of Eq. (1) as energies, although physically they correspond to deviations in the propagation constant, *β*, of the wavefunction, *ψ*, along the axial direction of the waveguides, with *k*_*z*_ = *k*_0_ + *β*, in which *k*_0_ = *n*_0_*ω/c* and *n*_0_ = 1.473 is the index of refraction of the surrounding glass. Thus, an adiabatic pump protocol can be written into a waveguide array by periodically modulating both the index of refraction and the separation in between the waveguides as a function of the propagation distance, *z*, which changes the effective on-site energy and the effective coupling coefficients, respectively^13^. A schematic of the experimental system is shown in Fig. 1a.

The adiabatic pump protocol that we choose is the Rice–Mele model^42^, which is a bipartite array generated by periodically modulating the on-site energies and coupling coefficients of a one-dimensional integer lattice with a two-member unit cell. The tight-binding Hamiltonian for the Rice-Mele model can be written as

2 $$\hat H_{{\mathrm{RM}}}\left( z \right) = \mathop {\sum}\limits_j {\left( {\frac{\tau }{2} + \frac{{\delta _j(z)}}{2}} \right)\left( {\hat c_j^\dagger \hat c_{j + 1} + {\mathrm{h}}.{\mathrm{c}}.} \right) + {\mathrm{\Delta }}_j(z)\hat c_j^\dagger \hat c_j}$$

in which $\hat c_j^\dagger$ and $\hat c_j$ are the creation and annihilation operators on lattice site *j*, *τ* is the uniform coupling strength, and *δ*_*j*_(*z*) and ∆_*j*_(*z*) are the degree of dimerization and staggered sublattice on-site energies, which are chosen to be periodic functions with period *Z*. Although the periodic on- and off-site modulations, *δ*_*j*_(*z*) and ∆_*j*_(*z*), are typically both chosen to be alternating sinusoids in the Rice–Mele model, the evanescent couplings between neighboring waveguides in our experiment instead result in a “lopsided” Rice–Mele system, with

3 $$\delta _j\left( z \right) = \tau \left( {e^{( - 1)^j\tilde \delta \,{\mathrm{sin}}\left( {\frac{{2\pi z}}{Z}} \right)} - 1} \right)$$

4 $${\mathrm{\Delta }}_j\left( z \right) = ( - 1)^j{\bar{\mathrm \Delta }}\,{\mathrm{cos}}\left( {\frac{{2\pi z}}{Z}} \right)$$

where ${\bar{\mathrm \Delta }}$ describes the degree of variation in on-site energy (as a result of the modulation of the index of refraction), and $\tilde \delta$ describes the degree of modulation of the coupling between the waveguides given a sinusoidal variation in the distance between them. For our experimental system with an average spacing between waveguides of *l* = 36 μm, a sinusoidal variation in the spacing of *δl* = ±8 μm, an average refractive index shift of ∆*n* = 2.7 × 10^−3^ from the surrounding glass substrate, and a sinusoidal variation in the refractive index of *δn* = ±0.3 × 10^−3^, the model coefficients are found to be *τ* = 0.55 cm^−1^, ${\bar{\mathrm \Delta }}/\tau$ = 6.97, and $\tilde \delta$ = 2.11 for *λ* = 1650 nm.

The topological properties of the Rice–Mele model are determined by the closed trajectory of the modulation in (*δ*_*j*_(*z*), Δ_*j*_(*z*))-space. If the system adiabatically evolves over a full period such that this trajectory encircles the origin, then a localized Wannier state arising from a given bulk band is transported to the right by a single unit cell (corresponding to Chern number 1). In terms of the edge properties, if an edge state is initially populated, then over the pump cycle it traverses the bulk and populates the edge state on the other side of the lattice. If the system adiabatically evolves over a full period such that its trajectory in (*δ*_*j*_(*z*), Δ_*j*_(*z*))-space does not contain the origin, then neither of these phenomena occur. We can confirm that our experimental pumping protocol realizes an adiabatic pump by simulating the instantaneous spectrum through a full modulation cycle and noting the appearance of edge states crossing the bulk bandgap, as shown in Fig. 1b.

There are two additional requirements for realizing quantized bulk transport in the Rice–Mele system. First, the initial state of the system must be a Wannier state and thus uniformly fill an entire bulk band of the system. Second, the modulation of the system must be slow enough to not introduce any inter-band couplings between the instantaneous eigenstates. Note that the requirement on the modulation speed of the Thouless pump is different for edge-to-edge transport and bulk transport. Thouless pump protocols preserve the system’s translational symmetry, and as such, different states within the same bulk band, which possess different momenta, cannot couple due to conservation of lattice momentum. Thus, realizing quantized bulk transport in a Thouless pump only requires bandgap-level adiabaticity as intraband transitions are prohibited. In contrast, to realize edge-to-edge transport, the system necessarily has broken translational symmetry, as both the initial and final states reside at the edges. As such, edge-to-edge pumping instead requires state-level adiabaticity.

In a perfectly clean waveguide array (i.e., one without disorder), we realize a near-Wannier initial state by choosing our input facet to coincide with the point in the modulation period where the waveguides are evenly spaced and have maximally detuned indices of refraction (i.e., on-site energies). At this point, an initial single-waveguide excitation has approximately uniform overlap with all of the instantaneous states of a single bulk band. We can confirm this feature numerically and demonstrate that our system is bandgap-level adiabatic through simulations with periodic boundary conditions using this single-waveguide input. In Fig. 1c, we show the nearly uniform and nearly constant projection of the propagating wavefunction onto the instantaneous eigenstates of the periodic system, |*φ*_*n*_〉, over a full modulation cycle. This simulation also confirms that although our system is only bandgap-level adiabatic, exhibiting very weak coupling between states in the upper and lower bulk bands, conservation of lattice momentum protects against intraband couplings, so the initial nearly uniform distribution of the wavefunction over a single bulk band is maintained through the complete modulation cycle.

As such, upon injecting this spatially localized wavefunction into the array and allowing it to propagate for a full adiabatic pump cycle, we experimentally observe the transport to be nearly a full unit cell, as shown in Fig. 1d, i.e., that

5 $${\mathrm{\Delta }}x_\psi \left( Z \right) = \left\langle {\psi (Z){\mathrm{|}}x{\mathrm{|}}\psi (Z)} \right\rangle - \left\langle {\psi (0){\mathrm{|}}x{\mathrm{|}}\psi (0)} \right\rangle \approx 1$$

In our waveguide arrays, the choice of which of the two waveguides in each unit cell to inject light into determines which bulk band of the system is excited, and as the two bulk bands possess opposite Chern numbers, this dictates the direction of propagation. In Fig. 1d, this displacement is calculated relative to the expected direction of travel for injection into each waveguide. Examples of the experimentally observed transport at the output facet of the waveguide array are shown in Fig. 1e.

**Fig. 2:**
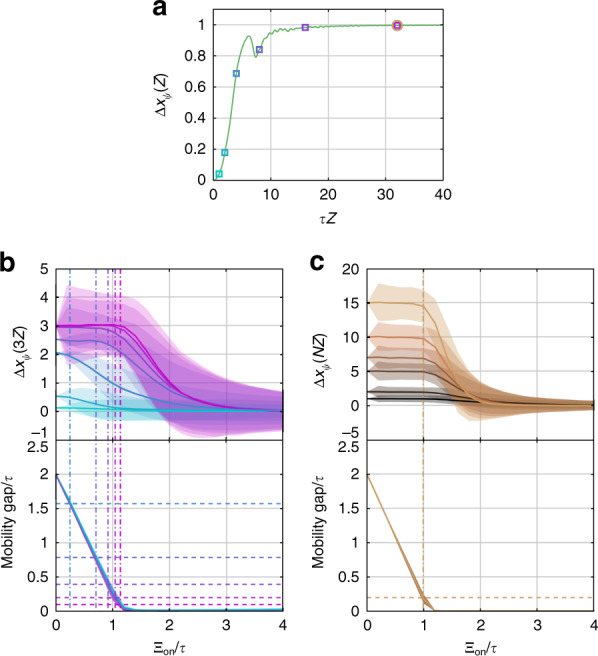
**a** Numerical calculation of Δ*x*_*ψ*_(*Z*) as a function of the product of the average coupling and modulation length, *τZ*, for a Rice–Mele system with sinusoidal variation of Δ_*j*_ and δ_*j*_. The color of the squares and circle corresponds to the values of *τZ* for the simulations shown in **b** (squares) and **c** (circle), below. **b** Ensemble-averaged displacement of a localized initial wavefunction (top panel) and minimum mobility gap (bottom panel) through three modulation periods as a function of the added on-site disorder, Ξ_on_. Different color curves correspond to different choices of modulation period, *τZ* = [1, 2, 4, 8, 16, 32, 64], listed from cyan to magenta. The shaded region behind each curve shows the range of ± one standard deviation of the ensemble. The vertical dash-dot lines running through both panels are a visual guide showing where the minimum mobility gap becomes equal to the modulation frequency for each choice of *τZ*. The horizontal dashed lines in the bottom panel indicate the modulation frequency, *Ω*. Note that for *τZ* = [1, 2], the modulation frequency is always greater than the system’s minimum mobility gap, even without any added disorder. **c** Similar to **b**, except that different color curves correspond to increasing numbers of modulation cycles, *N* = [1, 2, 5, 7, 10, 15], listed from black to light brown, for *τZ* = 32

For this experiment, the minimum pumping period required for bandgap-level adiabaticity is ~6 cm. For the Rice–Mele system to be bandgap-level adiabatic, the average coupling coefficient, *τ*, which sets the energy scale of the system, must yield a larger instantaneous bulk bandgap than the modulation frequency, *Ω* = 2*π/Z*. This amounts to requiring that the dimensionless product *τZ* be sufficiently large. This relationship can be effectively seen in Fig. 2a, where we numerically calculate Δ*x*_*ψ*_(*Z*) as a function of *τZ* and observe the appearance of a plateau, Δ*x*_*ψ*_(*Z*) ≈ 1, whose boundary is defined by *τZ* ≈ 20. Here, we use a sinusoidally modulated system, $\Delta _j\left( z \right) = ( - 1)^j\bar \Delta \,\cos (\Omega z)$, and $\delta _j\left( z \right) = ( - 1)^j\bar \delta \,\sin (\Omega z)$, and fix the ratios $\bar \Delta /\tau$ and $\bar \delta /\tau$ to maintain the same effective strength of the adiabatic pump.

Upon introducing disorder to the system, lattice momentum is no longer conserved, and for a finite system (with periodic boundary conditions), the bulk bands break up into two groups of *m* subbands each, where *m* is the number of unit cells. As long as the disorder is sufficiently weak that a bulk bandgap remains between these groups, the Chern number of the bands of the disordered system is well defined^2,3,39,40^. In a ground-state fermionic system (with all the subbands of a group equally populated), this topological invariant implies that the adiabatic pumping process will still yield quantized transport. However, in a nonequilibrium bosonic system without state-level adiabaticity, intra-subband transitions within a single band are now in principle possible, and these transitions can degrade the observed nearly quantized transport.

Nevertheless, despite the possibility for degraded transport properties in the presence of system disorder, we do not observe this to be a significant effect. To demonstrate that the nearly quantized transport seen in Thouless pumped photonic systems with only bandgap-level adiabaticity is tolerant to disorder, we add disorder terms to the lattice Hamiltonian, $\hat H = \hat H_{{\mathrm{RM}}} + \hat H_{{\mathrm{dis}}}$, with

6 $$\hat H_{{\mathrm{dis}}} = \mathop {\sum}\limits_j {{\mathrm{\Xi }}_{{\mathrm{on}}}\xi _{j,{\mathrm{on}}}\hat c_j^\dagger \hat c_j + {\mathrm{\Xi }}_{{\mathrm{off}}}\xi _{j,{\mathrm{off}}}\left( {\hat c_j^\dagger \hat c_{j + 1} + {\mathrm{h}}.{\mathrm{c}}.} \right)}$$

where Ξ_on_ and Ξ_off_ are the overall strength of the on- and off-site disorder, respectively, and $\xi _{j,{\mathrm{on}}},\xi _{j,{\mathrm{off}}} \in [ - 1,1]$ are uniformly distributed random numbers.

The nearly quantized bulk-band transport properties of the disordered Rice–Mele lattice can be seen in the top panels of Fig. 2b, c, in which the ensemble-averaged net displacement of a single-band spatially localized initial wavefunction exhibits a plateau until the strength of the disorder becomes sufficiently strong. Moreover, as seen in the bottom panels of Fig. 2b, c, the ensemble-averaged size of the minimum mobility gap between the bulk bands throughout the modulation cycle also decreases as the strength of the disorder is increased. By comparing the upper and lower panels of Fig. 2b, c, it is clear that the plateau terminates when the minimum mobility gap of the instantaneous eigenvalues becomes similar to the pump frequency, *Ω*. The ensemble of simulations shown here consists of 1000 independent realizations of the disorder and uses sinusoidal variations of the on- and off-site disorder coefficients. We have also numerically confirmed that the qualitative results are independent of the amount of diffraction of the propagating wavefunction, which is determined by ${\bar{\mathrm \Delta }}$ and $\bar \delta$. Thus, these simulations demonstrate that the presence of intraband transitions caused by the addition of disorder does not immediately result in the decay of the bulk transport properties. Instead, the average bulk transport in these systems remains nearly constant until the disorder is sufficiently strong to allow for Zener tunneling.

**Fig. 3:**
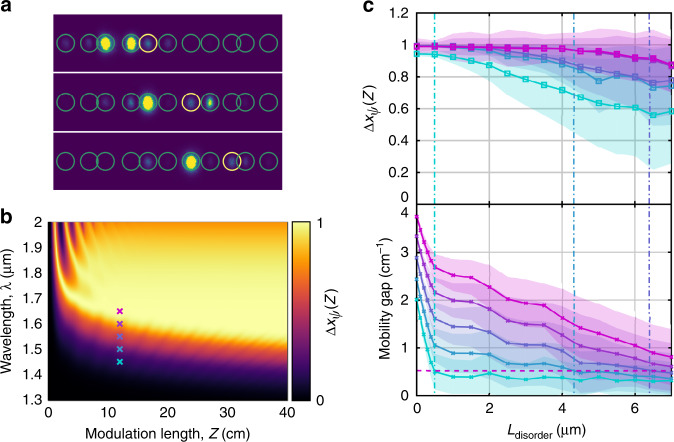
**a** Three exemplars of the direct observation of transport at the output facet of the waveguide array for *λ* = 1650 nm and *L*_disorder_ = 7 μm. The waveguide locations are outlined in green circles, except for the location of the initially excited waveguide, which is outlined in yellow. **b** Numerical calculation of Δ*x*_*ψ*_(*Z*) for the (ordered) Rice–Mele waveguide lattice, Eqs. (2)–(4), as a function of the wavelength, *λ*, and modulation period, *Z*. **c** Experimentally observed ensemble-averaged displacement (top panel) and simulated minimum mobility gap (bottom panel) versus the strength of the disorder for a single modulation cycle. Different colors correspond to different incident wavelengths, *λ* = [1450, 1500, 1550, 1600, 1650 nm], listed in order of cyan to magenta, and indicated in **b**. The modulation period is *Z* = 12 cm. The ensemble consists of 50 different realizations of the disorder. The shaded region behind each curve shows the range of ± one standard deviation of the ensemble. The modulation frequency, *Ω*, is indicated as a horizontal dashed line. Vertical dash-dot lines running through both panels are a visual guide showing where the minimum mobility gap becomes equal to the modulation frequency for each wavelength

To experimentally demonstrate that the bulk transport properties of this pumped system remain nearly quantized in the presence of system disorder while the system remains bandgap-level adiabatic, we add disorder to our waveguide arrays by displacing the average position of each waveguide by *L*_disorder_ *ξ*_*j*,off_, in which *L*_disorder_ is the magnitude of the shift in the center of each waveguide. Individual examples of the experimentally observed transport in the disordered waveguide array at the output facet are shown in Fig. 3a. To determine when the (ordered) waveguide array is bandgap-level adiabatic, we calculate Δ*x*_*ψ*_(*Z*) as a function of the wavelength and the modulation period, as shown in Fig. 3b. Here, we use the wavelength as an experimentally accessible proxy for the average coupling coefficient, *τ*, as the two are exponentially related, with longer wavelengths yielding larger average coupling coefficients. However, changing the wavelength also affects the other two model parameters, ${\bar{\mathrm \Delta }}$ and $\tilde \delta$, with longer wavelengths effectively decreasing both ${\bar{\mathrm \Delta }}/\tau$ and $\tilde \delta$, which ultimately results in decreasing the strength of the adiabatic pump. This is why the system leaves the bandgap-level adiabatic regime for increasing *λ* at fixed *Z*. The oscillations seen in Fig. 3b for short modulation cycles and long wavelengths correspond to when the system is in the “handoff” regime, i.e., Δ*x*_*ψ*_(*Z*) ≈ 1, not because the system is adiabatic but because the coupling strength and length conspire to completely transfer all of the light from one waveguide to its neighbor when the two are closest to each other during the modulation cycle. Likewise, the valleys between these peaks correspond to a similar effect where the coupling strength and length result in all of the light returning to the originally excited waveguide when the two waveguides are close together.

As seen by comparing the top and bottom panels in Fig. 3c, we observe a plateau in the ensemble-averaged transport that ends when the disorder becomes strong enough to close the instantaneous minimum mobility gap to be approximately equal to the modulation frequency, *Ω*. We note that in this experimental system, the plateau appears to terminate slightly before the numerical prediction, based on the size of the instantaneous mobility gap, would predict. This result is likely due to additional system disorder from fabrication imperfections. However, the observation that longer wavelengths, which are more adiabatic, exhibit longer transport plateaus as the disorder strength is increased confirms the expected qualitative behavior of the system. In addition, while the system remains bandgap-level adiabatic, not only does the ensemble-averaged transport remain such that Δ*x*_*ψ*_(*Z*) ≈ 1, but the distribution thereof remains narrow as well. This finding can also be seen in Fig. 3c, where the more adiabatic longer wavelengths (magenta colors) exhibit a significantly narrower distribution of their displacements than those for the shorter wavelengths, which have left the adiabatic regime (cyan colors).

## Discussion

In conclusion, we have experimentally demonstrated adiabatic pumping in a photonic system and shown that such systems can exhibit nearly quantized transport even if they are only bandgap-level adiabatic in the presence of disorder. Looking forward, it would be interesting to explore the connection between Thouless pumps and dynamical quantum ratchets^43,44^, as both types of systems can exhibit bulk transport due to modulation. In photonics, we envision that this expanded parameter space of temporally modulated systems, and in particular adiabatic pumps, can be used as a design principle for future on-chip slow-light applications.

## Materials and methods

A titanium:sapphire laser and amplifier system (Coherent, RegA 9000) with repetition rate 250 kHz, pulse duration 270 fs, and pulse energy 820 nJ was used to write the waveguide arrays into Corning Eagle XG borosilicate glass, *n*_0_ = 1.473. To control the size and shape of the laser’s focal volume within the glass sample, the beam was first sent through a beam-shaping cylindrical telescope and then focused with a ×50 aberration-corrected microscope objective (NA = 0.55). Physically, the waveguides were written by translating the glass sample through the laser’s focus using a set of high-precision three-axis stages (Aerotech, model ABL20020). The speed of this translation was varied between 9 and 27 mm/s to generate refractive index shifts between ∆*n*_0_ = 3.0 × 10^−3^ and ∆*n*_0_ = 2.4 × 10^−3^, noting that the slower speeds correspond to larger increases in the refractive index of the system. This range of variation in the index of refraction of the waveguides, as well as the average separation and variation in the waveguide spacing, *l* = 36 μm and *δl* = 8 μm, respectively, were chosen to allow for the system to be in the bandgap-level adiabatic regime for our longest excitation wavelength of *λ* = 1650 nm, while being in the non-adiabatic regime for our shortest excitation wavelength, *λ* = 1450 nm.

The waveguide arrays were measured by butt-coupling a single-mode optical fiber to a single waveguide in the array at the input facet of the glass sample. A tunable mid-infrared diode laser, 1450–1650 nm (Agilent 8164B), was used as the light source for these measurements. Light was collected at the output facet of the glass sample using an NA = 0.2 microscope objective lens and imaged onto a near-infrared InGaAs camera (ICI systems).

In numerical simulations, these waveguides were modeled as possessing a hyper-Gaussian distribution to their index of refraction,

7 $${\mathrm{\Delta }}n\left( {x,y,z} \right) = {\mathrm{\Delta }}n_0\left( z \right)e^{ - \left( {\left( {\frac{x}{{\sigma _x}}} \right)^2 + \left( {\frac{y}{{\sigma _y}}} \right)^2} \right)^3}$$

Here, ∆*n*_0_(*z*) is the *z*-dependent shift in the index of refraction of the waveguide from the surrounding glass, and *σ*_*x*_ = 3.2 μm and *σ*_*y*_ = 4.9 μm characterize the elliptical geometry of the waveguide.

## Data Availability

The data that support the findings of this study are available from the corresponding authors on reasonable request.

## Code availability

The code that support the findings of this study are available from the corresponding authors on reasonable request.
